# Heated egg white has no effect, but lactic fermented and unheated egg white reduces abdominal fat in rats

**DOI:** 10.1186/s12944-019-1133-1

**Published:** 2019-10-26

**Authors:** Ryosuke Matsuoka, Yayoi Takahashi, Ayano Muto, Mamoru Kimura

**Affiliations:** R & D Division, Kewpie Corporation, Sengawa Kewport, 2-5-7, Sengawa-cho, Chofu-shi, Tokyo, 182-0002 Japan

**Keywords:** Egg white protein, Abdominal fat, Diet, Heated, Lactic fermented, Micelle, Rats

## Abstract

**Background:**

We previously reported the abdominal fat-reducing effect of unheated egg white proteins (EWP); however, unheated egg white is actually rarely consumed. We thus investigated the effect of heated egg white on abdominal fat in rats.

**Methods:**

Male SD rats were divided into two groups that were allowed to consume different dietary preparations containing casein or heated egg white for 4 weeks (Trial 1). We studied whether a heated form and a lactic fermented form of egg white (FLE) are as effective as unheated egg white for reducing abdominal fat. For this, we divided male SD rats into four groups that were allowed to consume different dietary preparations containing casein, unheated egg white, heated egg white, or lactic fermented egg white for 4 weeks (Trial 2).

**Results:**

Animals in the heated egg white group showed no significant difference in abdominal fat weight compared with those in the casein group (Trial 1). Animals in the unheated egg white group and the FLE group had significantly lower levels of abdominal fat weight than those in the casein group (Trial 2). Ovalbumin in heated egg white was degraded by pepsin, whereas ovalbumin in unheated egg white and lactic acid fermented egg white was not degraded appreciably by pepsin. It was reported that EWP inhibit triglyceride absorption in rat. In the present study, EWP pepsin hydrolysate inhibited the micellar solubility of fatty acids in vitro. In particular, ovalbumin inhibited the micellar solubility of fatty acids.

**Conclusions:**

These results indicate that lactic fermented egg white reduces visceral fat in rats and suggest that different levels of susceptibility of ovalbumin to pepsin digestion underlie the varying effectiveness among the egg white preparations.

## Background

Egg white proteins are a fat-free, high-protein food. They include many essential amino acids, have an amino acid score of 100, and are high-quality sources of proteins combined with proteins derived from milk and soybeans [1]. Egg white proteins have been reported to promote iron absorption and lower blood cholesterol [2, 3]. They have also been reported to increase the body protein mass of rat carcasses [4]. Previously, we reported that egg white proteins increased the body protein mass in rats and reduced their body fat and visceral fat [5]. Although unheated egg white was used in previous studies, egg white is actually far more likely to be consumed in a heated form. However, to the best of our knowledge, the effects of the consumption of heated egg white on visceral fat have not been studied.

In previous studies evaluating net protein utilization rates of egg white preparations, we reported that unheated, soft-boiled, and heated preparations of egg white retain high levels of net protein utilization, which are even significantly higher than the rate for whey proteins [6].

Egg white proteins have a visceral fat-reducing effect, but it is difficult to enjoy the effect by drinking egg white itself because of its flavor and physical properties. Therefore, we have developed lactic acid fermented egg white [7], a flavor-improved egg white product prepared by lactic acid fermentation. However, the effects of lactic acid fermented egg white on the visceral fat weight remain to be elucidated.

Metabolic syndrome is considered to be a risk factor for arteriosclerotic diseases [8], as it causes impaired glucose tolerance, dyslipidaemia, and hypertension via increased visceral fat. If an actually edible form of egg white reduces visceral fat, such a preparation can be tested in clinical studies and may be applied to the prevention and mitigation of metabolic syndrome in the future.

In the present study, we investigated whether heated and lactic acid fermented preparations of egg white are as effective as unheated egg white for the reduction of visceral fat.

## Methods

### Materials

Ovalbumin (OA), Ovotransferrin (OT), ovomucoid (OM), and lysozyme (LY) were purchased from Sigma-Aldrich co. LLC (St. Luice, USA) The unheated EWP used were desiccated egg white K type (CS No. 2; Kewpie Egg Corporation, Tokyo, Japan). Casein was purchased from Oriental Bioservice, Inc. (Kyoto, Japan). Heated EWP were prepared from unsterilized egg white (Kewpie Egg Corporation, Tokyo, Japan) that had been heated at 95 °C for 10 min. Lactic fermented egg white (FLE) was obtained from Kewpie Egg Corporation (Tokyo, Japan) [7]. Heated EWP and FLE were freeze-dried and crushed uniformly. Protein contents were determined using the Dumas method [9] and were casein 85.9%, unheated EWP 86.1%, heated EWP 82.7%, and FLE 39.8%.

### Animals and diets

In this study, 4-week-old SD male rats (90–120 g) were used. They were kept in stainless steel cages under conditions of light cycle 08:00–20:00, temperature 23 ± 1 °C, and humidity 50 ± 2%.

The test feed was adjusted in accordance with AIN-76 feed composition [10]: protein (casein or unheated EWP, heated EWP, FLE) 20%, β cornstarch 10%, cellulose 5%, mineral mix (AIN-76) 3.5%, vitamin mix (AIN-76) 1%, corn 10%, and choline bitartrate 0.25%, brought up to 100% with sucrose. In Trial 2, Casein, unheated egg white, heated egg white, and FLE were added in a manner such that the resulting nitrogen contents were equalized. Specifically, casein 20.0 g/100 g, unheated egg white 20.0 g/100 g, heated egg white 20.1 g/100 g, and FLE 43.2 g/100 g were added.

Rats were fed each diet for 4 weeks using a pair-feeding protocol. At the end of the study period, the rats were made to fast for 8 h and, under Nembutal anesthesia (Dainippon Sumitomo Pharma Co., Ltd., Tokyo, Japan), were killed by aortic blood collection; the liver and epididymal, mesenteric, and perirenal adipose tissues were then collected. Blood was centrifuged at 3000 rpm for 10 min, and the extracted serum was stored at − 80 °C until use.

Animal experiments were conducted under the guidelines for animal experiments at Kewpie Corporation (Tokyo, Japan) and Law no. 105 and Notification no. 6 of the Government of Japan. The authorization number is 18–1. Animal experiments were performed from 5 January 2010 to 9 February 2010 (Trial 1), and from 11 September 2009 to 9 October 2009 (Trial 2).

### Analysis

Triglyceride, Phospholipids, Free fatty acid, Glucose, ALT and AST in serum were determined using Triglyceride C-Test Wako, Phospholipids E-Test Wako, NEFA C-Test Wako, Glucose CII Test Wako, Transaminase CII Test Wako, respectively (Wako Co. Ltd., Tokyo, Japan). Insulin and leptin concentrations were determined using Morinaga Insulin ELISA Kit and Morinaga Leptin ELISA Kit, respectively (Morinaga Institute of Biological Science, Inc., Kanagawa, Japan). Concentrations of triglycerides and phospholipids in the carcass and liver were measured by chemical protocols after extracting the lipids with the Folch method [11–13].

### Preparation of casein, EWP, and EWP constituent protein hydrolysates

A total of 10 g of casein, EWP, OA, OT, OM, and LY was hydrolyzed by 400 mL of experimental gastric juice [0.1% (w/v) pepsin in 100 mM KCl] with HCl at pH 2.0 and 37 °C for 24 h [14]. The reaction mixtures were adjusted to a pH of 7.0 with 1 M KOH, freeze-dried, and powdered to obtain casein pepsin hydrolysate (C-ph), EWP pepsin hydrolysate (EWP-ph), OA pepsin hydrolysate (OA-ph), OT pepsin hydrolysate (OT-ph), OM pepsin hydrolysate (OM-ph), and LY pepsin hydrolysate (LY-ph).

### SDS-page

The degree of hydrolysis of unheated EWP, heated EWP, and FLE was determined by SDS-PAGE. The SDS-PAGE samples were incubated at 95 °C for 5 min using a thermal cycler, and 5 μg of protein from each sample was then electrophoresed on a 5%–20% gradient polyacrylamide gel. The gel was stained using Coomassie Brilliant Blue.

### Preparation of micellar solutions

Micellar solutions containing 6.6 mM sodium taurocholate, 0.6 mM phosphatidylcholine, 1.0 mM oleic acid in 132 mM NaCl, and 15 mM sodium phosphate buffer (pH 6.8) were prepared by sonication and maintained at 37 °C for 24 h to ensure stabilization of the micelles [14].

### Micellar solubility of free fatty acids in vitro

The micellar solubility of fatty acids was measured in accordance with the methods described by Matsuoka et al. A solution containing C-ph, EWP-ph, OA-ph, OT-ph, OM-ph, and LY-ph was added to the micelles. The samples were added at a final concentration of 20 mg/mL to the micelles, whereas the reconstituted EWP-ph was added at a final concentration of 16 mg/mL to the micelles. In accordance with a previous study, the reconstituted EWP-ph included 54% OA-ph, 13% OT-ph, 11% OM-ph, and 3.5 LY-ph. The samples were incubated at 37 °C for 1 h and passed through a 0.2 μm syringe filter [14]. Free fatty acid levels in the filtrates were analyzed using the method described by Tsujita et al. [15].

### Statistical analysis

Test results are shown as mean ± SEM. Comparison between two groups was conducted using Student’s *t*-test. Comparison among three or more groups was conducted using Tukey’s test. A *p-*value less than 0.05 were considered statistically significant. For statistical analysis, the software Dr. SPSS for Windows (SPSS Co. Ltd.) was used.

## Results

### Effect of heated EWP on abdominal fat in rats (Trial 1)

No significant differences in body weight increase, food intake, abdominal fat weight, serum triglyceride, serum free fatty acid, and hepatic phospholipids were found between the two groups (Table 1). Serum phospholipid and hepatic triglyceride concentrations in the heated egg white group were significantly lower than those in the casein group (Table 1).

**Table 1:** Effects of Heated EWP on abdominal fat, serum, and hepatic lipid parameters in rats (Trial 1)

	Casein	Heated EWP	Change (%) vs. Casein
Growth Parameter (g/day)			
Body weight gain	6.64 ± 0.13	6.74 ± 0.20	98.6 ± 1.4
Food intake	16.3 ± 0.0	16.0 ± 0.0	101 ± 3
Adipose Tissue Weight (g/100 B.W.)			
mesenteric	1.03 ± 0.08	1.05 ± 0.11	102 ± 11
perirenal	1.63 ± 0.17	1.72 ± 0.19	106 ± 12
epididymal	1.44 ± 0.15	1.51 ± 0.15	105 ± 10
total	4.10 ± 0.10	4.39 ± 0.39	105 ± 10
Serum Parameter			
Triglyceride (mg/100 mL)	64.1 ± 12.2	85.3 ± 13.7	133 ± 21
Phospholipid (mg/100 mL)	118 ± 7	159 ± 11*	135 ± 9
Free fatty acid (mEQ/L)	0.680 ± 0.073	0.774 ± 0.071	114 ± 10
Hepatic lipids (mg/g liver)			
Triglyceride	28.1 ± 2.3	17.7 ± 1.4*	63.1 ± 5.0
Phospholipid	37.9 ± 1.2	34.5 ± 0.6	90.9 ± 1.6

Mean ± SE of 8 rats. * Significant difference at *p* < 0.05 by Stndent’s *t*-test

*EWP* egg white protein

### Effects of unheated or heated lactic fermented EWP on abdominal fat in rats (Trial 2)

No significant differences in body weight increase and food intake were found among the four groups. The mesenteric visceral fat weight in the FLE group was significantly smaller than that in the casein group. Pararenal fat weight and paratesticular fat weight in the unheated egg white group and the lactic acid fermented egg white group were significantly smaller than those in the casein group, but the weight of neither type of fat differed significantly between the heated egg white group and the casein group. The total weight of mesenteric fat, pararenal fat, and paratesticular fat was significantly smaller in the heated egg white group and lactic acid fermented egg white group than in the casein group, but no significant difference was observed between the heated egg white group and the casein group (Table 2).

**Table 2:** Effects of unheated EWP, heated EWP, or FLE on the abdominal fat, serum, and hepatic lipid parameters in rats (Trial 2)

	Casein	Unheated EWP	Heated EWP	FLE	Change (%) vs. Casein		
					Unheated EWP	Heated EWP	FLE
Growth Parameter (g/day)							
Body weight gain	6.91 ± 0.10	6.44 ± 0.26	7.17 ± 0.16	6.34 ± 0.28	93.2 ± 3.8	103 ± 2	91.7 ± 4.0
Food intake	16.2 ± 0.2	15.7 ± 0.4	16.3 ± 0.2	15.5 ± 0.5	97.1 ± 2.2	101 ± 1	95.9 ± 3.4
Adipose Tissue Weight (g/100 B.W.)							
mesenteric	0.738 ± 0.074^a^	0.503 ± 0.036^ab^	0.725 ± 0.053^a^	0.427 ± 0.057^b^	71.1 ± 5.2	97.8 ± 5.7	58.1 ± 5.6
perirenal	1.80 ± 0.04^a^	1.07 ± 0.01^b^	1.72 ± 0.15^a^	0.854 ± 0.100^b^	63.7 ± 5.6	89.1 ± 7.8	49.4 ± 4.3
epididymal	1.51 ± 0.05^a^	1.03 ± 0.10^b^	1.76 ± 0.08^a^	1.05 ± 0.08^b^	72.5 ± 6.5	109 ± 7.9	69.1 ± 4.0
total	4.06 ± 0.08^a^	2.82 ± 0.22^b^	4.20 ± 0.21^a^	2.33 ± 0.02^b^	68.3 ± 5.1	98.3 ± 6.3	58.3 ± 3.6
Serum Parameter							
Triglyceride (mg/100 mL)	114 ± 29^a^	72.7 ± 7.3^ab^	131 ± 23^a^	35.4 ± 5.0^b^	63.7 ± 6.4	114 ± 20	31.1 ± 4.4
Phospholipid (mg/100 mL)	159 ± 7	184 ± 7	218 ± 17	121 ± 8	115 ± 4.0	137 ± 11	76.3 ± 5.2
Free fatty acid (mEQ/L)	0.816 ± 0.093^a^	0.773 ± 0.076^ab^	0.782 ± 0.054^ab^	0.531 ± 0.036^b^	94.6 ± 9.3	95.7 ± 6.5	64.9 ± 4.4
Insulin (ng/mL)	3.13 ± 0.46	2.67 ± 0.51	3.36 ± 0.77	1.72 ± 0.33	85.4 ± 16.2	108 ± 25	54.9 ± 10.6
Leptin (ng/mL)	2.19 ± 0.17^ab^	1.64 ± 0.15^a^	2.94 ± 0.57^b^	1.29 ± 0.18^a^	77.2 ± 6.7	134 ± 26	58.7 ± 8.2
Hepatic Lipids (mg/g liver)							
Triglyceride	18.2 ± 1.8^a^	15.1 ± 1.5^ab^	19.4 ± 1.6^a^	12.9 ± 1.7^b^	83.2 ± 7.0	107 ± 8	71.0 ± 8.1
Phospholipid	27.2 ± 0.7	26.9 ± 0.5	24.8 ± 0.7	27.6 ± 1.0	98.6 ± 1.9	91.0 ± 2.7	100 ± 4

Mean ± SE of 8 rats. ^ab^: Different letters show a significant difference at *p* < 0.05 by Turkey’s test., *EWP* egg white protein, FLE: lactic fermented egg white

Serum and liver triglyceride concentrations and serum free fatty acid concentrations in the lactic acid fermented egg white group were significantly lower than those in the casein group, but the unheated egg white group and the heated egg white group showed no significant differences in these parameters compared with the casein group. Serum leptin levels in the unheated egg white group and the lactic acid fermented egg white group were significantly lower than the level in the heated egg white group, but did not differ significantly from the level in the casein group. No significant differences among the four groups were observed in serum and liver phospholipid concentrations or serum insulin levels (Table 2).

### Pepsin susceptibility of proteins in different egg white preparations

**Fig. 1:**
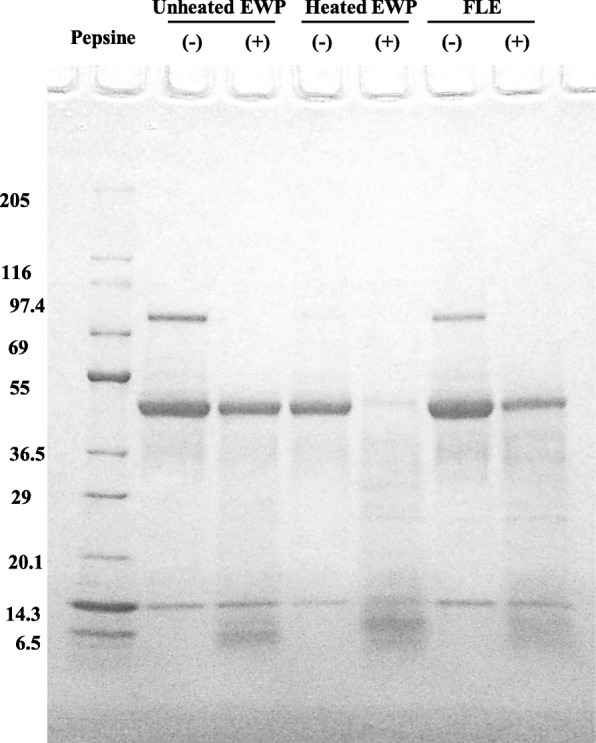
Electrophoresis of unheated EWP, heated EWP, FLE, and pepsin hydrolysates. EWP: egg white protein, FLE: lactic fermented egg white

Pepsin digestion products were prepared from unheated egg white, heated egg white, and lactic acid fermented egg white to compare their digestibility. The results showed that ovalbumin in unheated egg white and lactic acid fermented egg white underwent no appreciable pepsin digestion, whereas ovalbumin in heated egg white was degraded by pepsin (Fig. 1).

### Effects of EWP pepsin hydrolysate on the micellar solubility of fatty acids

EWP-ph significantly inhibited the micellar solubility of free fatty acids to a greater extent than C-ph. OA-ph, OT-ph, and LY-ph significantly inhibited the micellar solubility of free fatty acids compared with C-ph. Furthermore, there was no significant difference between EWP-ph and reconstructed EWP (Table 3). The pepsin responsiveness could not be calculated in this study, but it may be necessary to determine this in a future study.

**Table 3:** Effects of protein hydrolysate on the micellar fatty acid contents in vitro

	Micellar fatty acid (%)
Casein-PH	33.9 ± 1.1
EWP-PH	28.9 ± 1.4*
Casein-PH	49.1 ± 0.9
Ovalbumin-PH	36.3 ± 0.7^#^
Ovotransferrin-PH	36.0 ± 0.6^#^
Ovomucoid-PH	65.5 ± 6.8
Lysozyme-PH	32.0 ± 0.3^#^
Casein-PH	38.8 ± 0.6
Reconstract EWP-PH	38.1 ± 0.6

Mean ± SE of 3–5 samples. Different letters were shown a significant difference (*p* < 0.05). *EWP*: egg white protein, *PH*: pepsin hydrolysate, Reconstructed EWP: Ovalbumin-PH: Ovotransferrin-PH: Ovomucoid-PH: Lysozyme-PH = 54:12.5:11:3.5

## Discussion

The results from this study showed that the visceral fat weight in the lactic acid fermented egg white group was significantly smaller than that in the casein group, as was the case for the unheated egg white group.

The egg white component responsible for visceral fat reduction is unlikely to be an amino acid, since heated egg white did not show the effect of reducing visceral fat like unheated and lactic acid fermented preparations of egg white did, despite the fact that all of the three preparations had an identical amino acid composition. Thus, the effect appeared to be attributable to a peptide or a polymer. It was previously reported that unheated egg white is hardly digested by pepsin, whereas heated egg white is readily digested by it [16]. The results from the present study showed that unheated egg white, but not heated egg white, reduced visceral fat. Therefore, if ovalbumin in the lactic acid fermented egg white, which was demonstrated to reduce visceral fat in the present study, is less susceptible to pepsin digestion, differences in pepsin digestibility may be involved in the visceral fat-reducing effect. Indeed, ovalbumin in the lactic acid fermented egg white was confirmed to be far less susceptible to pepsin digestion in this study. These results demonstrated the possibility that the visceral fat reduction was mediated by the egg white protein ovalbumin in a pepsin-resistant form. Furthermore, we calculated donate (%) of Egg white component for reducing micellar solubility of fatty acids. The results shown that 55.8% (OA), 13.0% (OT), 7.88% (OM), 3.87% (LY), and 19.4% (Others). These results indicated that main component of egg white protein is OA.

We previously reported that egg white proteins suppress the absorption of triglycerides in rats subjected to thoracic duct lymph cannulation surgery [13]. The mechanisms underlying the observed suppressive effect on triglyceride absorption include inhibition of the lipase activity by ovalbumin [17] and binding of ovalbumin to free fatty acids produced by the action of lipases. Although the results were obtained with egg white proteins, high levels of water-holding capacity, settling volume, and relative viscosity were noted [13], suggesting that the visceral fat weight was reduced as a result of the suppression of triglyceride absorption by reducing the fatty acid content in bile acid micelles (Table 3). Further, as the heated egg white preparation, which contains ovalbumin in a digestible form, did not show the visceral fat-reducing effect in the present study, a major part of the visceral fat-reducing effect of egg white proteins is presumably attributable to different degrees of degradation of ovalbumin by pepsin in the stomach. It was reported that heated egg white did not inhibit lipid (cholesterol) absorption in hamsters [18].

After the egg white protein consumption in this study, fat absorption was reduced by about 20% [13], whereas the visceral fat weight decreased by about 30%. Thus, the reduced fat absorption may account for the majority, but not the entirety, of the visceral fat reduction effect of egg white. Therefore, it is possible that an unknown peptide that is in a pepsin-resistant form in unheated egg white and lactic acid fermented egg white, but that is in a pepsin-susceptible form in heated egg white, reduces visceral fat.

In addition to the suppression of fat absorption, possible mechanisms of visceral fat reduction also include i) enhanced β-oxidation of fatty acids in the liver, ii) reduced synthesis of fatty acids in the liver, and iii) reduced adipocyte size through the action on visceral fat. Previous studies showed that egg white proteins increased the activity of enzymes related to β-oxidation in the liver [5]. We confirmed that egg white proteins decrease the enzyme activity related to fatty acid synthesis in the liver [5]. We also confirmed that egg white proteins decrease the size of adipocytes [5]. In previous studies, it was thought that these three actions was confirmed to occur after the consumption of egg white protein.

Dietary proteins reported to decrease visceral fat include β-conglycinin from soybeans, which decreases visceral fat by reducing fat absorption [19]. Lactoferrin from milk has also been reported to reduce visceral fat through signal transduction by acting on visceral adipocytes [20]. Egg white proteins presumably decrease visceral fat through both mechanisms of fat absorption suppression and direct action on visceral fat; it has been inferred that the main mechanism is the suppression of fat absorption.

The daily intake of 5 g of soybean β-conglycinin has been reported to decrease visceral fat in humans [21]. In contrast, lactoferrin has been shown to reduce visceral fat at a daily intake of 300 mg [22]. Given the visceral fat-reducing mechanism of egg white that is assumed to be mainly mediated by the suppression of fat absorption, the effective amount is presumably close to that of β-conglycinin, and visceral fat reduction is likely to be achieved with the responsible component ovalbumin at a daily intake of 5 g. However, the present study has shown that other egg white components, such as ovotransferrin and lysozyme, decrease the fatty acid content in bile acid micelles (Table 3). To consume 5 g of these fractions per day, 8 g of egg white protein needs to be consumed per day. This amount, which is equivalent to 2.5 eggs, is difficult to consume in the form of raw egg white. About 125 g of lactic acid fermented egg white is equivalent to 8 g of egg white proteins and appeared to be a realistic daily intake as lactic acid fermented egg white is a beverage with a yogurt flavor. We reported lactic fermented egg white (EWP content was 8 g/day) reduced visceral fat area in human [23]. The crude protein quantities were matched among the four groups in this study. If they were not, the FLE group would have received a lower amount of dietary protein and shown a weaker effect of visceral fat reduction. In a previous study, we evaluated the lowest effective dose of egg white protein in humans, and have reported that 8 g of egg white protein reduced more visceral fat than 6 g of egg white [24].

Whole egg includes many component prevent from arteriosclerotic diseases [25]. Since visceral fat obesity is a risk factor for arteriosclerosis. In this experiment, we show that arteriosclerotic diseases may be prevented and improved by consuming lactic acid fermented egg white. However, even though it has physiological activity, it is not easy to decrease visceral fat solely via dietary means. Therefore, the use of lactic acid fermented egg white would need to be combined with lifestyle improvements.

## Conclusions

In conclusion, the data from the present study demonstrate that lactic acid fermented egg white reduced the visceral fat weight in rats. As for the mechanism underlying this, the distinct susceptibility of ovalbumin in lactic acid fermented egg white was shown to be responsible for the effect.

## Data Availability

All experiments generated or analysed in this study are included in this manuscript.

## Abbreviations

EWP: egg white protein
FLE: lactic fermented egg white
LY: Lysozyme
OA: Ovalbumin
OM: Ovomucoid
OT: Ovotransferrin
Ph: pepsine hydrolysate
SD: Sprague-dewley
